# Bi-portal Endoscopic Calcaneoplasty for Haglund's Deformity

**DOI:** 10.7759/cureus.62658

**Published:** 2024-06-18

**Authors:** Abhishek Saini, Shubham Srivastava, Abhishek Agarwal, Ravindra Mohan, Abhinav Vatsa

**Affiliations:** 1 Department of Sports Medicine, King George's Medical University, Lucknow, IND; 2 Department of Orthopedic Surgery, Prasad Institute of Medical Sciences, Lucknow, IND; 3 Department of Orthopedic Surgery, King George's Medical University, Lucknow, IND; 4 Department of Orthopedics, Government Medical College, Jalaun, IND

**Keywords:** haglund’s deformity, fowler philip angle, endoscopy, calcaneoplasty, aofas score

## Abstract

Introduction

Haglund’s deformity is an abnormal bony postero-superior calcaneal prominence which causes the posterior heel pain. Surgery is the choice of treatment after failed conservative management. Both open and endoscopic techniques are used to treat this condition. In this article, we discuss endoscopic calcaneoplasty for the management of such cases.

Methods

All included patients underwent bi-portal endoscopic calcaneoplasty. Clinical outcomes were assessed by using the visual analog scale (VAS) score and the American Orthopaedic Foot and Ankle Society (AOFAS) score. The radiological outcome was assessed by a change in the Fowler-Philip angle (FPA). All patients were followed at one month, three months, and six months postoperatively.

Results

A total of 22 patients were included in this prospective study. Patients were followed up to six months postoperatively. The mean VAS score was 6.32 ± 0.65 which was significantly reduced to 0.91 ± 0.68 (p < 0.001) at six months. Similarly, AOFAS Score was improved to 90.01 ± 2.67 (p < 0.001) from 64.36 ± 7.07 preoperatively. The mean Fowler Philip Angle was reduced from 72.45° ± 3.74° to 65.77° ± 2.25° at six months (p < 0.001).

Conclusion

Bi-portal endoscopic calcaneoplasty significantly improves clinical outcomes in Haglund’s deformity. Compared to open procedures, bi-portal endoscopic calcaneoplasty offers several advantages, including shorter recovery times, smaller incisions, and better clinical results for Haglund's deformity.

## Introduction

Haglund's deformity, also known as "pump bump," or “calcaneal exostosis” is a painful condition that affects the posterior side of the heel. It is characterized by the presence of a bony enlargement of the calcaneum, which can lead to irritation of the Achilles tendon and discomfort during daily activities.

Although recent studies raised suspicion on the commonly accepted idea that Haglund deformity frequently correlates with insertional Achilles tendinopathy, this hypothesis is still widely believed [1-3].

Haglund's syndrome affects 25% of those with insertional Achilles tendinopathy. Physical attritions are the primary cause of the tendinopathy and mechanical damage of the calcaneum. According to a hypothesis, the anterior Achilles insertion pushes on the bony prominence, especially if the gastroc-soleus-Achilles myotendinous complex is tight or contracted, causing localized injury to the tendon including longitudinal tears in the tendon. The hind foot varus can also result in the calcaneus impinging on the calcaneus in patients with cavovarus foot [3].

Traditional surgical approaches to address this condition often involve open procedures with longer recovery times and potential complications. However, in recent years, the advent of minimally invasive techniques, such as bi-portal endoscopic calcaneoplasty, has shown promise in improving outcomes, reducing patient morbidity and early return to daily activities [4,5].

In this article, we will explore the clinical and radiological outcomes of bi-portal endoscopic calcaneoplasty in the management of Haglund's deformity [6].

We will discuss the methodology of the study, the results obtained from a cohort of 22 patients, and the implications of these findings for the treatment of this common foot condition.

## Materials and methods

This prospective study was conducted at the King George's Medical University, Lucknow, a tertiary-level teaching medical university in India. A total of 25 patients with a clinical diagnosis of Haglund's deformity were included in this study. Written and informed consent were obtained from all patients included in this study. Ethical clearance was obtained from the Institutional Ethical Committee, King George's Medical University, Lucknow (Approval No.: 102nd ECM IIB/P117). All those patients who presented with symptoms of pain, swelling, and tenderness at the posterior aspect of the heel were considered. Only the patients who had taken at least six months of conservative treatment with no response were included in this study. The diagnosis was confirmed through physical examination. Radiographic imaging (X-rays) of the weight-bearing lateral view of the ankle joint were obtained. X-ray showed a prominent bursal projection of the calcaneum. To supplement the diagnosis, magnetic resonance imaging (MRI) was done to assess the extent of deformity, Achilles, and soft tissue involvement [2,3].

Surgical procedure

**Figure 1:**
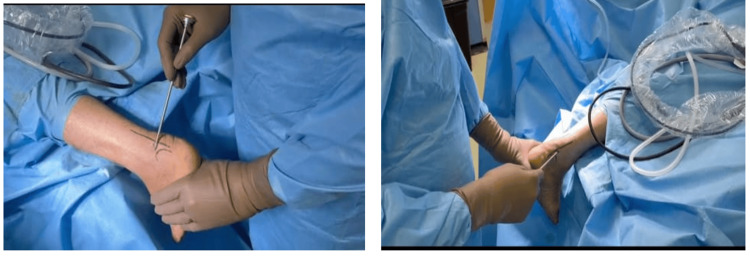
Picture showing lateral and medial ankle portals respectively.

All patients underwent bi-portal endoscopic calcaneoplasty as the primary surgical intervention. The procedure was performed under regional block or spinal anesthesia. The patient was taken into a prone position. We have used bi-portal, i.e., lateral and medial endoscopic portals. No tourniquet was used. Bony landmarks were marked while keeping the ankle in a neutral position. The technique involved two small incisions (portals) made on either side of the Achilles tendon at the site of the Haglund's deformity. The posterolateral portal was made, 2 cm proximal to the tip of the lateral malleolus medial to peroneal tendons and lateral to the Achilles tendon, and the posteromedial portal was made just medial to the Achilles tendon (Figure 1).

**Figure 2:**
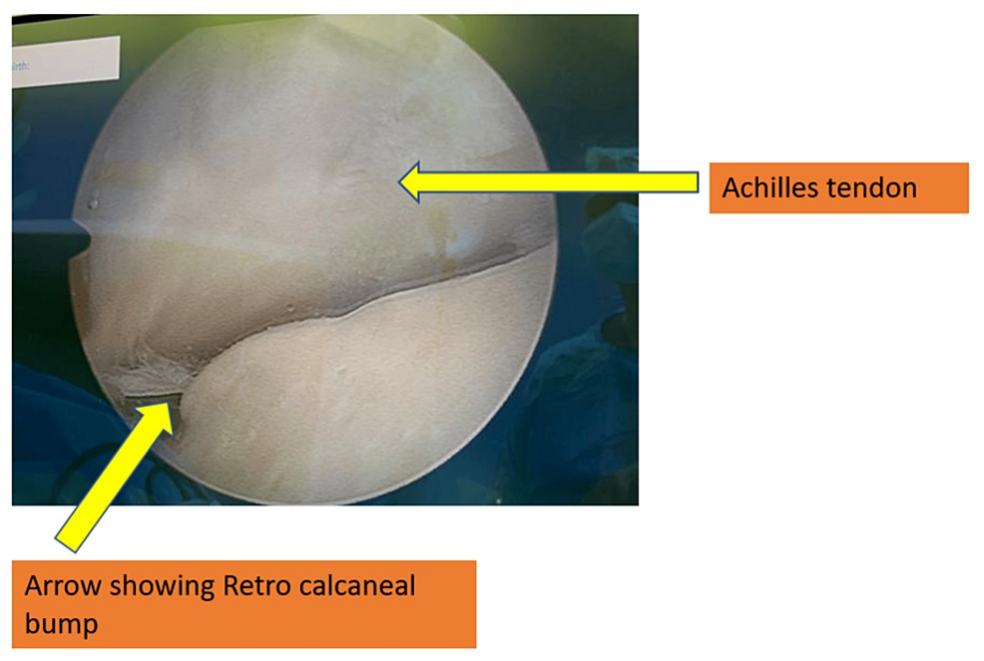
Endoscopic view of retro-calcaneal region.

**Figure 3:**
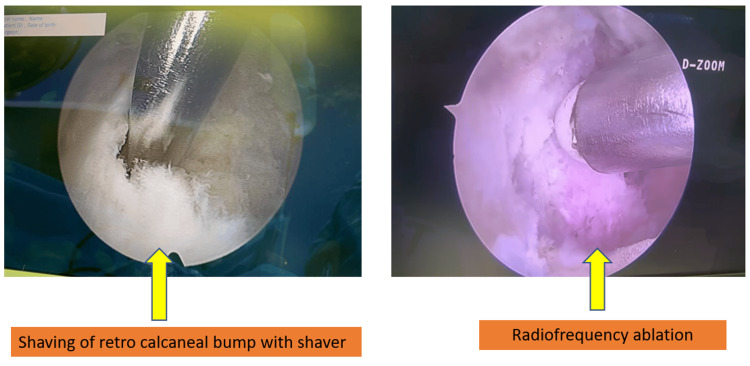
Shaving and radio-frequency ablation of the retro-calcaneal bump.

A stab incision was made and spread with mosquito forceps. Entry into retro-calcaneal space was achieved. The retro-calcaneal bursa was shaved off using a shaver. Both the medial and lateral corners of Haglund’s bump were visualized on the camera screen. With the access from the portal, an arthroscopic burr was inserted and bump and sharp edges were removed. Radiofrequency ablation was utilized for control of bleeding, if any. However, it is important not to leave any sharp bony prominence on either side of the postero-superior calcaneal tubercle during endoscopic calcaneoplasty. Fluoroscopic confirmation was done. The adequacy of bone excision is confirmed by the absence of impingement with the ankle in full dorsiflexion (Figures 2, 3).

Outcome measures

The American Orthopaedic Foot and Ankle Society (AOFAS) score, assesses the function of the foot and ankle, taking into account factors including pain, alignment, and patient satisfaction. Higher scores indicate better function and lesser pain. Scores range from 0 to 100 [7]. The Visual Analog Scale (VAS) score is a self-reported measure of pain. On a 10-point scale, 0 represents no pain and 10 represents the worst suffering that can be imagined, patients indicate how much pain they are experiencing [8].

The degree of calcaneal resection attained during surgery is evaluated by the radiographic measurement, Fowler-Philip angle (FPA). A more significant decrease in the bony prominence is indicated by a greater reduction in the FPA [9].

Patients were followed up at regular intervals for up to six months postoperatively. During these visits, AOFAS scores, VAS scores, and FPA measurements were recorded to track changes in function, pain levels, and radiographic outcomes. Complications, if any, were documented.

Patients were followed up at regular intervals for up to six months postoperatively. During these visits, AOFAS scores, VAS scores, and FPA measurements were recorded to track changes in function, pain levels, and radiographic outcomes. Complications, if any, were documented.

## Results

Patient demographics

The study group consisted of 25 patients with diagnosed cases of Haglund's deformity. Three patients were lost in follow-up. A total of 22 patients were considered for the study. There were ten males and twelve females, with an average age of 44.18± 10.94 years (range: 28 to 65 years). All patients had failed conservative treatments, including rest, physical therapy, and nonsteroidal anti-inflammatory drugs (NSAIDs), prior to undergoing bi-portal endoscopic calcaneoplasty (Table 1).

**Table 1:** Age-wise distribution of patients.

Age intervals	N	%
25-30 years	2	9.1
31-40 years	7	31.8
41-50 years	7	31.8
51-60 years	3	13.6
More than 60 years	3	13.6
Total	22	100

The preoperative mean AOFAS score was 64.36 ± 7.07. At one month postoperatively, the mean AOFAS score significantly improved to 80.73 ± 4.07 (p < 0.001; N=22). This improvement in function and pain continued through the six-month follow-up, with a mean AOFAS score of 90.01 ± 2.67 (p < 0.001). Notably, all patients reported satisfaction with their outcomes at the final follow-up (Table 2).

**Table 2:** VAS score. VAS: Visual analog scale.

VAS	Mean	SD	Median	Min	Max	Valid N	P value
Preoperative value	6.32	.65	6.00	5.00	7.00	22	< 0.001
After 1 month	3.50	.51	3.50	3.00	4.00	22	< 0.001
After 3 months	2.32	.48	2.00	2.00	3.00	22	< 0.001
After 6 months	.91	.68	1.00	.00	2.00	22	< 0.001

The preoperative mean VAS score for pain was 6.32 ± 0.65. After one month postoperatively, the mean VAS score significantly decreased to 3.50 ± 0.51 (p < 0.001; N=22). This reduction in pain persisted at the six-month follow-up, with a mean VAS score of 0.91 ± 0.68 (p < 0.001) (Table 3).

**Table 3:** AOFAS score. AOFAS: American Orthopaedic Foot and Ankle Society.

AOFAS	Mean	SD	Median	Min	Max	Valid N	P value
Preoperative value	64.36	7.07	65.00	51.00	75.00	22	< 0.001
After 1 month	80.73	4.07	80.00	75.00	88.00	22	< 0.001
After 3 months	85.59	7.53	86.50	54.00	92.00	22	< 0.001
After 6 months	90.00	2.67	90.00	86.00	95.00	22	< 0.001

A radiographic assessment of the FPA revealed a significant reduction in the bony prominence. The preoperative mean angle was 72.45° ± 3.74°. Postoperatively, the mean angle reduced to 65.77° ± 2.25° at six months (p < 0.001, N=22). These results indicated a substantial correction of the deformity (Table 4).

**Table 4:** Fowler-Philip angle (FPA).

FPA	Mean	SD	Median	Min	Max	Valid N	P value
Preoperative value	72.45	3.74	72.00	66.00	78.00	22	< 0.001
After follow-up at 6 months	65.77	2.25	66.00	63.00	69.00	22	< 0.001

No major complications, such as wound infections, nerve injuries, or Achilles tendon injuries, were observed in any of the 22 patients. Minor complications, including transient numbness around the incision site and mild swelling, were reported by two patients but resolved spontaneously within the first month after surgery.

## Discussion

Haglund's deformity has a significant impact on a patient's mobility, everyday activities, and general well-being. It is defined by a bony protrusion at the posterior part of the heel. Patients are frequently urged to seek medical care due to the persistent pain and functional restrictions. Although open surgical techniques have historically been the basis of care, they have intrinsic disadvantages such as significant disruption of soft tissue, extended recovery times, and increased risk of complications [10].

The treatment of Haglund's deformity has changed significantly with the introduction of minimally invasive procedures like bi-portal endoscopic calcaneoplasty. Small incisions and sophisticated endoscopic equipment are incorporated into this method, which has numerous advantages over open surgery. Early return to routine activities is encouraged, discomfort following surgery is minimized, rehabilitation is accelerated, and soft tissue structures are preserved [11]. Additionally, the lower incidence of infections and wound complications improves patient satisfaction and safety.

Our study contributes to the growing pool of data showing the safety and effectiveness of bi-portal endoscopic calcaneoplasty for Haglund's deformity. The noteworthy enhancements noted in pain, function, and radiography results highlight the practical advantages of this minimally invasive technique. The treatment improves patients' quality of life by reducing symptoms and restoring normal foot biomechanics by treating the underlying bony prominence and retrocalcaneal impingement [12].

The fact that our study revealed a high degree of patient satisfaction is evidence of the extent to which bi-portal endoscopic calcaneoplasty helps alleviate the pain and limitations in function caused by Haglund's deformity. Patient-reported outcome measures, like the VAS and AOFAS scores, offer insightful information about the actual improvements individuals undergoing this surgery actually experience. Moreover, the reduction of bone prominence as evidenced by radiography validates the anatomical effectiveness of the procedure, ensuring sustained symptom alleviation and delaying the advancement of the illness [7].

Though our study demonstrates that bi-portal endoscopic calcaneoplasty produces encouraging results, there are certain considerations to remain in view. In order to evaluate the durability of results and determine the risk of illness recurrence, larger-scale studies with longer-term follow-up are important, as shown by the relatively small sample size and short follow-up period. Additional study is also required to explore patient-specific factors affecting treatment results and compare the efficacy of open versus minimally invasive procedures [13].

## Conclusions

Bi-portal endoscopic calcaneoplasty emerges as a promising surgical technique for the management of Haglund's deformity. In our study, this minimally invasive procedure demonstrated significant relief in pain, improved function, and a substantial reduction in the bony prominence associated with Haglund's deformity. Besides simple mechanical decompression of the retro-calcaneal space, endoscopic calcaneoplasty can remove the degenerated posterior calcaneal wall cartilage that may also be a source of insertional pain. These positive outcomes were sustained up to six months postoperatively, with no major complications reported.

The other advantages of bi-portal endoscopic calcaneoplasty over traditional open procedures include smaller incisions, reduced soft tissue disruption, faster recovery times, and a lower risk of complications. In summary, bi-portal endoscopic calcaneoplasty represents a valuable addition to the surgical options available for Haglund's deformity, offering patients significant relief from pain and improved walking ability. While this study presents encouraging results, further research with larger sample sizes and longer follow-up periods is warranted to confirm the durability of these outcomes.
